# Modified Application of the Abdominal Re-Approximation Anchor Device in the Closure of Septic Open Abdomen in a Patient With Class III Obesity

**DOI:** 10.7759/cureus.58749

**Published:** 2024-04-22

**Authors:** Daryn Nguyen, Jiali Tan, Christie Bialowas

**Affiliations:** 1 Department of Plastic and Reconstructive Surgery, Albany Medical College, Albany, USA; 2 Department of Plastic and Reconstructive Surgery, Albany Medical Center, Albany, USA

**Keywords:** damage control laparotomy, class iii obesity, primary fascial closure, wound closure technique, abdominal closure, abra, fascial closure, open abdomen surgery

## Abstract

The Abdominal Re-Approximation Anchor (ABRA^®^) is a pivotal dynamic wound closure system utilized for achieving primary fascial closure in patients undergoing open abdomen surgeries. However, its efficacy can be hindered in patients with class III obesity due to anatomical complexities and compromised tissue characteristics. Here, we present the unique case of a 25-year-old woman with class III obesity (body mass index (BMI) ≥ 40 kg/m^2^) who required primary abdominal closure following complications of an ileostomy repair. Traditional placement of the ABRA device was not feasible due to thick subcutaneous tissue layers. Consequently, a modified application of ABRA was decided based on clinical judgment, whereby the ABRA button anchors were strategically placed internally under the subcutaneous tissue instead of externally on the skin surface. The patient completed six intraoperative tightenings of the ABRA device via this novel technique and was treated with washouts over the course of two months until complete resolution was achieved. The presented case demonstrates a successful modification of the ABRA wound closure device to suit an open abdomen patient with class III obesity.

## Introduction

Abdominal Re-Approximation Anchor (ABRA®) is a dynamic wound closure device that has shown efficacy in achieving primary fascial closure in patients with an open abdomen (OA) following damage control laparotomy [1]. Procedures such as the damage control laparotomy that involves the maintenance of an open abdomen are a necessary process in the management of severe intra-abdominal injury [2]. In some cases, primary abdominal wall closure following damage control is not possible, necessitating a different approach for these patients, especially in the face of bowel edema and loss of domain. Standard abdominal wall closure following these procedures has been accomplished through numerous methods, including mesh repair (MR), component separation (CS), and delayed primary closure techniques (DPC), through direct sutured fascial re-approximation, but such techniques when performed alone have been shown to be fraught with complications [3]. One meta-analysis identified 26 studies of total abdominal closure using MR, CS, and DPC and found estimates for abdominal complications to be 41%, 17%, and 17%, respectively, with mortality estimates of 6% for cases of DPC alone and 0.5% for MR alone [3].

In septic patients with severe peritonitis and OA treatment, delayed dynamic fascial closure combined with negative-pressure vacuum-assisted closure (VAC) has been shown to significantly decrease morbidity, mortality, and complications such as the incidence of incisional hernias [4]. The ABRA device is presently the only dynamic fascial traction device commercially available, and it is employed to facilitate fascial apposition in delayed OA closures [1,5].

Modifications of the ABRA dynamic closure system are rarely employed but are occasionally necessary to achieve appropriate fascial closure when patient anatomy compromises traditional ABRA closure techniques. One modified technique such as the U-shaped button anchor placement has seen success in achieving fascial re-approximation and closure in six OA cases complicated by severe peritonitis [6]. In this paper, we present a case of a severely septic open abdomen in a patient with class III obesity (body mass index (BMI) ≥ 40 kg/m^2^), with a BMI of 68.4 kg/m^2^, in which the ABRA device button anchors were placed internally in the subcutaneous space above the abdominal fascia instead of externally on the surface of the skin.

## Case presentation

**Figure 1:**
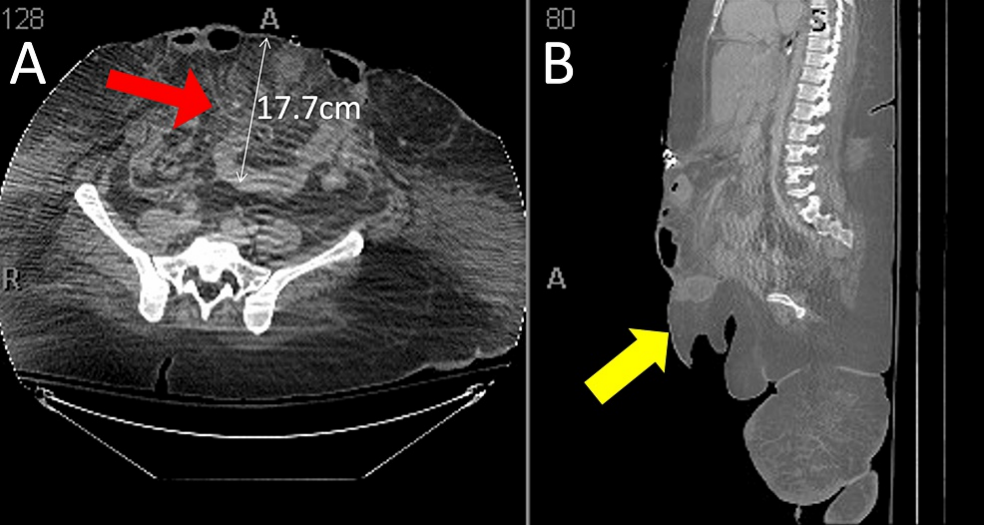
Preoperative computed tomography (CT) scans of the patient's abdomen and bowel herniation. A) Axial CT image of the pelvis showing a 17.7cm deep herniation (red arrow) of the small and large bowel protruding through the muscular abdominal wall and subcutaneous adipose tissue. B) Sagittal CT image showing the bowel herniation (yellow arrow) through the abdominal wall and hanging below the pubic symphysis.

A 25-year-old female with a BMI of 68.4 kg/m^2^ presented with rectal injury following sexual assault. She was initially treated with laparoscopic loop ileostomy and repair of the vaginal and rectal injuries. This was complicated by retraction of the ostomy and difficulty with the output control due to her significant body habitus. She underwent ileostomy reversal six weeks after the initial surgery due to the difficulties with the ostomy and healing of the rectal lacerations. She developed an anastomotic bleed a few days postoperatively, necessitating evacuation of the clot, resuscitation, and creation of end ileostomy. There was a complete disruption of the anastomosis at the ileostomy site, and due to this anastomotic leakage, the omentum had become ischemic. A resection of the distal small bowel was required. This resulted in an OA to allow for further abdominal washouts and stabilization of the patient. Consultation for abdominal reconstruction occurred 19 days from the initial takeback. She had a frozen abdomen and loss of domain with a 17.7 cm deep defect and most of her abdominal contents herniating through her muscular abdominal wall and hanging below her pubic symphysis outside the rectus muscles (Figure 1).

A modified technique of ABRA application

**Figure 2:**
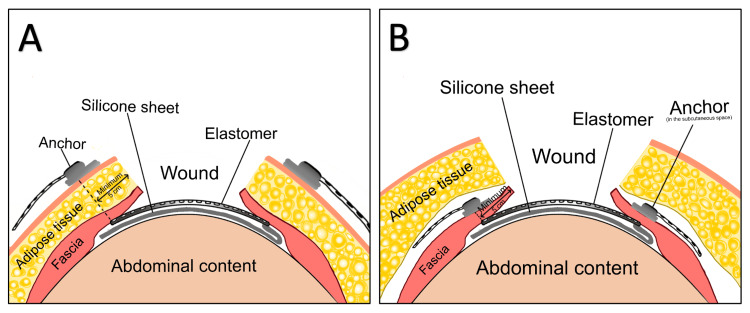
Schematic illustrations of traditional versus modified ABRA applications in the closure of open abdomens A) Traditional ABRA application with anchors placed external to the surface of the skin. The elastomers extend through the skin, subcutaneous adipose tissue, and rectus fascia. B) Modified ABRA application with anchors placed subcutaneously on the abdominal fascia, as done for the presented case. The elastomers extend through the rectus fascia only, and the tails are left in the subcutaneous space between the adipose tissue and the fascial layer. ABRA: Abdominal Re-Approximation Anchor Image Credits: Daryn Nguyen

Due to the significant loss of domain and the large scale of the defect, we planned on utilizing the ABRA device to aid in the delayed closure of her abdomen. However, her subcutaneous tissue was nearly 20 cm (about 7.87 in) thick, and this would not allow for adequate pressure on the muscle wall with the button anchors to achieve primary closure. As a result, we lifted subcutaneous planes over the abdominal fascia and placed the ABRA elastomers through the rectus fascia only. The buttons were placed against the fascial wall as well, leaving the device completely in the subcutaneous space instead of over the skin as traditionally anchored (Figure 2).

**Figure 3:**
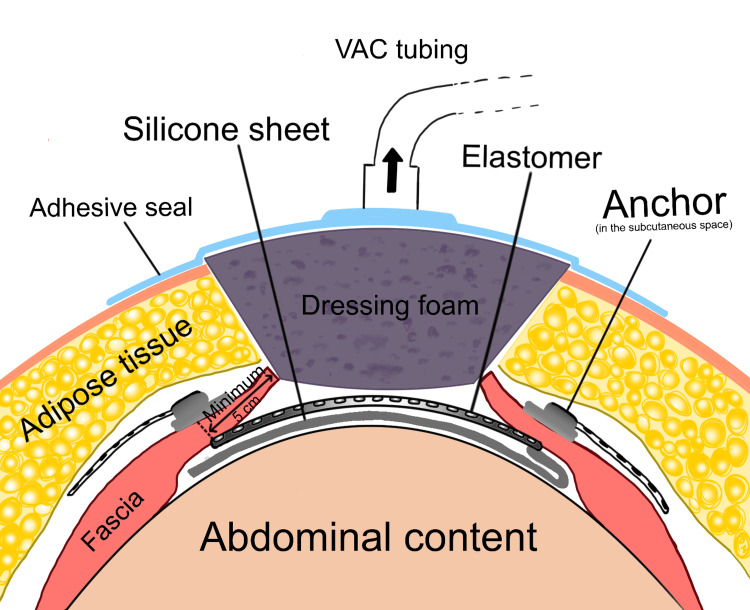
Schematic illustration of ABRA application with wound vacuum-assisted closure (VAC). This VAC dressing was placed over the entire defect. The patient underwent six intraoperative tightenings of the ABRA elastomers, each time bringing the fascial edges closer together. Between each tightening, the wound VAC dressing was replaced to maintain the sterility of the wound. ABRA: Abdominal Re-Approximation Anchor Image Credits: Daryn Nguyen

**Figure 4:**
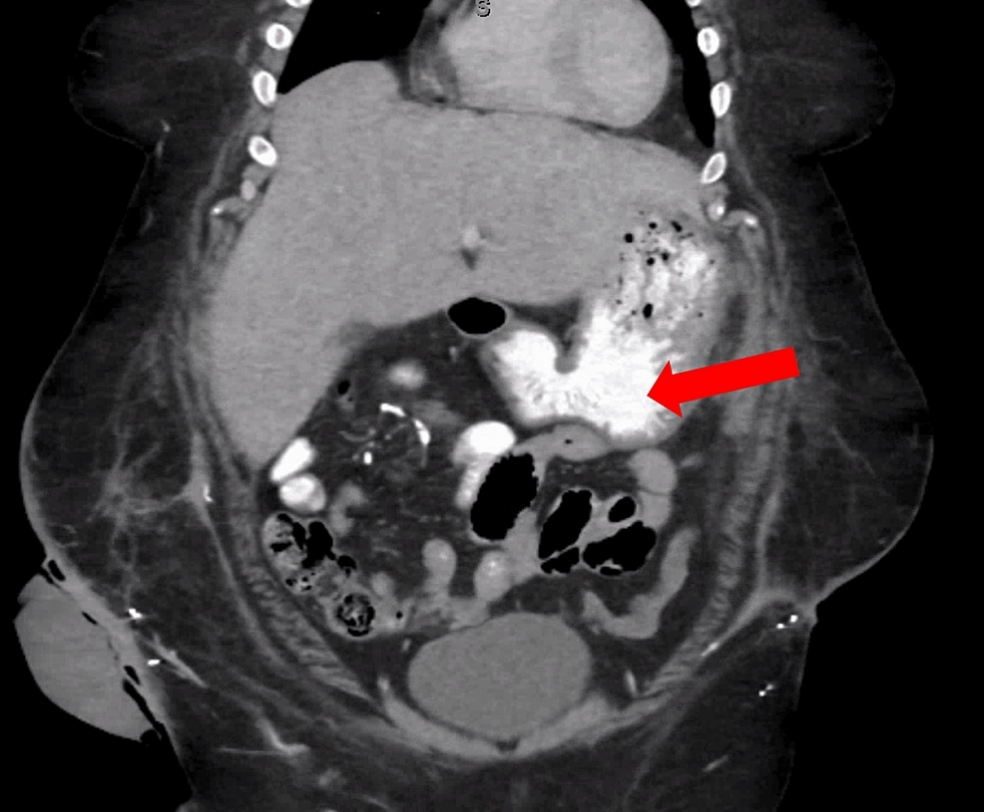
Postoperative CT scan of the abdomen with intravenous (IV) contrast showing the absence of a gastric fistula Coronal CT image of the abdomen taken two months following primary abdominal closure with the ABRA device showing enteric contrast (red arrow) in the stomach. There is no leakage of contrast into the peritoneum, indicating that the gastric fistula resolved, likely spontaneously. ABRA: Abdominal Re-Approximation Anchor

Following the standard of care, as laid out by the device’s manufacturing guidelines, we placed a wound vacuum-assisted closure (VAC) over the entire defect (Figure 3). The patient was then taken to the operating room every three to four days for tightening of the device internally. Six intraoperative tightenings of the ABRA elastomers were completed and complicated by feculent ostomy spillage and a gastric fistula. The cause of the fistula did not appear to involve the ABRA silicone sheet. We hypothesize that the ileostomy repair led to the formation of adhesions between the stomach and abdominal wall, which were ultimately disrupted during the re-approximation of the abdomen and resulted in the development of the gastric fistula. Once the rectus muscles were approximated enough, bilateral rectus muscle flaps were used for closure via an anterior component separation. Due to the width of the defect, we determined the anterior component separation was necessary to prevent the risk of abdominal compartment syndrome. She was then taken every few days for the management of the fistula and control of the high-output ostomy leakage, which were both managed surgically by extensive washout and debridement of the abdominal wall. The gastric fistula was finally controlled after further washouts over two months, and abdominal closure was achieved. A repeat computed tomography (CT) scan showed no further gastric fistula, which likely resolved spontaneously, and a competent abdominal wall (Figure 4), and six months later, she had her ostomy reversed with no complications. Her abdominal closure was found to be intact at that time, and she was able to be primarily closed without difficulty.

## Discussion

Open abdominal (OA) surgery on patients with obesity presents unique challenges due to factors such as altered anatomy, reduced tissue oxygenation, and compromised immune systems. Anatomically, patients with obesity have thicker subcutaneous tissue and larger abdominal adipose tissue deposits, which delay wound healing and impede wound closure [7]. Additionally, co-morbidities such as hypertension and type II diabetes mellitus further increase surgical risks and impair healing time [7].

Numerous techniques to achieve temporary closure in OA patients exist. Besides the ABRA dynamic closure system, these include static traction devices, such as the Wittmann Patch, as well as older techniques such as the Bogota bag, component separation techniques, and the vacuum-pack technique [4,5,8]. However, these methods may cause damage to the fascial edges and often fail to address the issue of fascial retraction, complicating successful closure [8,9]. For complex OA patients with obesity, primary fascial closure via these techniques is associated with higher rates of re-operation due to poor skin closure and surgical site infection [6,10,11].

The ABRA dynamic wound closure system offers advantages in addressing lost abdominal domain and achieving complete primary fascial closure, even in complex OA cases [5,6,12]. These advantages can be attributed to the fact that the wound edges are able to be steadily re-approximated over a gradual period of time by adjusting the device’s elastomers to graduated markings on a regular basis, thereby mitigating issues of fascial retraction [5]. However, the system faces limitations when implemented on patients with obesity for several reasons [11]. Reports in the literature detail ABRA applications in individuals with obesity, with worse outcomes, including longer ICU stays, higher rates of requiring mechanical ventilation, and lower primary closure rates when compared to patients who were not obese [10,11]. Despite this, dynamic closure with the ABRA device may be the best choice for patients with obesity to avoid complications from other closure techniques. Currently, there is no study of ABRA being used on patients with class III obesity with BMIs over 50 kg/m^2^.

Achieving fascial closure using standard ABRA application is exceptionally difficult in a patient with class III obesity. In the standard approach, the button anchors are placed externally over the abdomen skin, and the elastomer tails pass through the fascia, adipose, and skin layers. Thick subcutaneous tissue, such as in our presented case (20 cm), prevents the ABRA dynamic closure system from reaching an angle that would provide optimal appositional traction and pressure for the muscle planes. In this patient, we placed the ABRA components internally, directly over the rectus fascia in the subcutaneous space thereby bypassing the thick subcutaneous adipose tissue and allowing for more efficient distribution of force needed for primary fascial closure. To our best knowledge, this modified approach toward ABRA-assisted dynamic closure in a patient with class III obesity is the first application of its kind.

## Conclusions

In cases of severe intra-abdominal injuries, such as those with septic OAs where traditional wound closure methods fall short, the ABRA device has emerged as a promising solution, especially for large fascial defects. However, traditional applications of the ABRA device are limited by patient anatomy and body habitus. The thickness of the subcutaneous adipose layers in patients with class III obesity prevents an adequate distribution of force needed for primary fascial closure. As presented in this case, we circumvented this anatomical barrier by submerging the button anchors below the subcutaneous tissue and placing the elastomers through the rectus fascia. This modification was successful and may be a promising innovation for achieving fascial closure in other cases of septic OA patients with class III obesity.
